# 9-Benzyl-6-benzyl­sulfanyl-9*H*-purin-2-amine

**DOI:** 10.1107/S1600536814001986

**Published:** 2014-02-12

**Authors:** Maywan Hariono, Habibah A. Wahab, Mei Lan Tan, Mohd Mustaqim Rosli, Ibrahim Abdul Razak

**Affiliations:** a Pharmaceutical Design and Simulation (PhDs) Laboratory, School of Pharmaceutical Sciences, Universiti Sains Malaysia, 11800 Minden, Pulau Pinang, Malaysia; b Malaysian Institute of Pharmaceuticals and Nutraceuticals, Ministry of Science Technology and Inovation, 11700 Halaman Bukit Gambir, Pulau Pinang, Malaysia; c X-ray Crystallography Unit, School of Physics, Universiti Sains Malaysia, 11800 USM, Penang, Malaysia

## Abstract

In the title compound, C_19_H_17_N_5_S, the dihedral angles between the purine ring system (r.m.s. deviation = 0.009 Å) and the S-bound and methyl­ene-bound phenyl rings are 74.67 (8) and 71.28 (7)°, respectively. In the crystal, inversion dimers linked by pairs of N—H⋯N hydrogen bonds generate *R*_2_^2^(8) loops. C—H⋯N inter­actions link the dimers into (100) sheets.

## Related literature   

For background to the biological activity of thio­purine derivatives, see: Hadda *et al.* (2009 ▶); Nguyen *et al.* (2009 ▶). For further synthetic details, see: Banh *et al.* (2011 ▶); Salvatore *et al.* (2002 ▶, 2005 ▶).
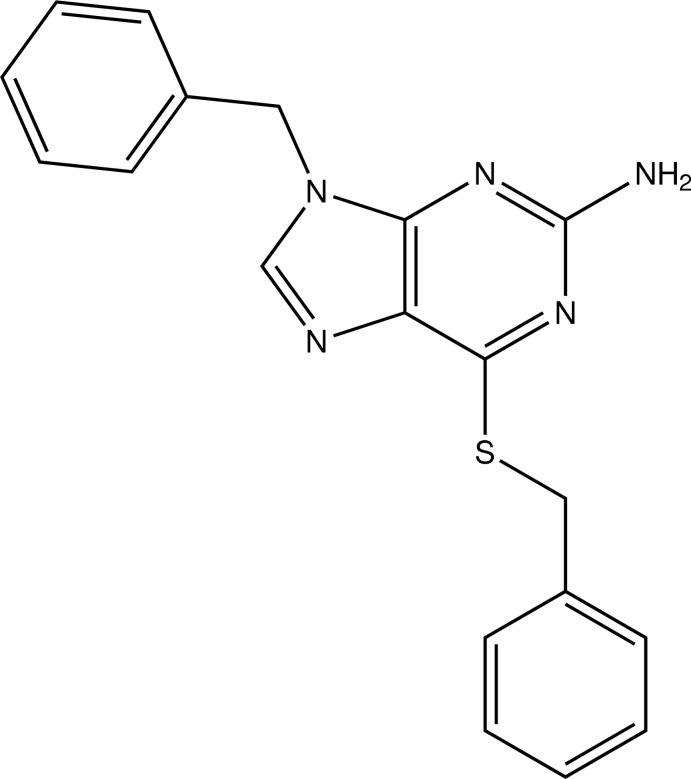


## Experimental   

### 

#### Crystal data  

C_19_H_17_N_5_S
*M* *_r_* = 347.44
Monoclinic, 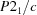

*a* = 16.7346 (7) Å
*b* = 5.5511 (3) Å
*c* = 20.4817 (10) Å
β = 121.325 (3)°
*V* = 1625.31 (14) Å^3^
*Z* = 4
Mo *K*α radiation
μ = 0.21 mm^−1^
*T* = 100 K
0.69 × 0.19 × 0.14 mm

#### Data collection  

Bruker SMART APEXII CCD diffractometer
Absorption correction: multi-scan (*SADABS*; Bruker, 2009) ▶ *T*_min_ = 0.868, *T*_max_ = 0.972
16728 measured reflections
4956 independent reflections
3416 reflections with *I* > 2σ(*I*)
*R*_int_ = 0.048

#### Refinement  

*R*[*F* ^2^ > 2σ(*F* ^2^)] = 0.050
*wR*(*F* ^2^) = 0.137
*S* = 1.06
4956 reflections
234 parameters
H atoms treated by a mixture of independent and constrained refinement
Δρ_max_ = 0.33 e Å^−3^
Δρ_min_ = −0.58 e Å^−3^

### 

Data collection: *APEX2* (Bruker, 2009) ▶; cell refinement: *SAINT* (Bruker, 2009) ▶; data reduction: *SAINT*; program(s) used to solve structure: *SHELXTL* (Sheldrick, 2008 ▶); program(s) used to refine structure: *SHELXTL*; molecular graphics: *SHELXTL*; software used to prepare material for publication: *SHELXTL* and *PLATON* (Spek, 2009 ▶).

## Supplementary Material

Crystal structure: contains datablock(s) I. DOI: 10.1107/S1600536814001986/hb7176sup1.cif

Structure factors: contains datablock(s) I. DOI: 10.1107/S1600536814001986/hb7176Isup2.hkl

Supporting information file. DOI: 10.1107/S1600536814001986/hb7176Isup3.cml

CCDC reference:

Additional supporting information:  crystallographic information; 3D view; checkCIF report

## Figures and Tables

**Table 1:** Hydrogen-bond geometry (Å, °)

*D*—H⋯*A*	*D*—H	H⋯*A*	*D*⋯*A*	*D*—H⋯*A*
N5—H1*N*5⋯N3^i^	0.91 (3)	2.14 (3)	3.040 (3)	173 (2)
C7—H7*B*⋯N3^ii^	0.99	2.57	3.548 (2)	172
C8—H8*A*⋯N2^iii^	0.95	2.39	3.274 (2)	155

Symmetry codes: (i) 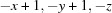; (ii) 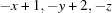; (iii) 
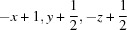
.

**Fig. 1.**
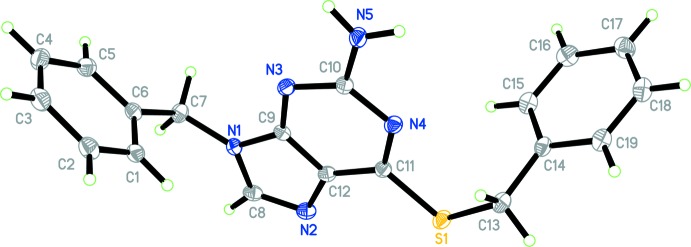
The asymmetric unit of the title compound, showing 50% probability displacement ellipsoids.

**Fig. 2.**
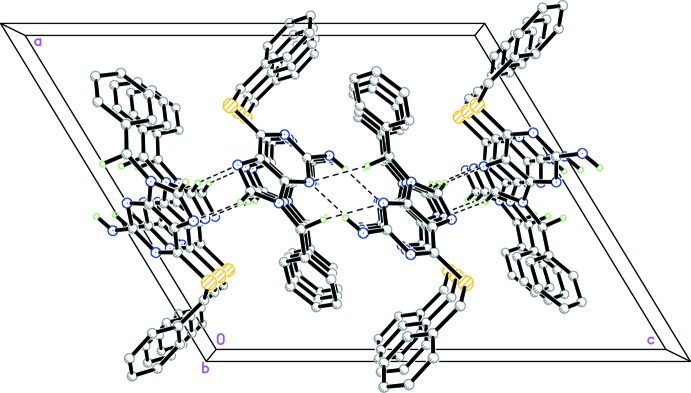
The crystal packing of (I). Dashed lines indicate hydrogen bonds. H atoms not involved in the hydrogen bond interactions have been omitted for clarity.

## supplementary crystallographic information

### 1. Introduction

Thio­purine and its analogues possess a broad pharmacological activity for example as a cytotoxic agent (Nguyen *et al.*, 2009) and in the treatment of lupus nephritis (Hadda *et al.*, 2009). As part of our studies in this area, we report the synthesis and structure of the title compound.

### 2. Experimental

The method to synthesize the title compound was modified from a few papers (Banh *et al.*, 2011; Salvatore *et al.*, 2002; Salvatore *et al.*, 2005). 2-amino-9H-purine-6-thiol (0.598 mmol) was mixed with cesium carbonate (0.598 mmol) in 3.5 ml of di­methyl­formamide and then stirred vigorously for 15 minutes. Another mixture containing benzyl bromide (1.315 mmol), tetra­butyl­ammonium iodide (0.598 mmol) in 3.5 ml of DMF was added to the first mixture and the stirring was continued at room temperature for six hours. The reaction progress was monitored by TLC using *n*-hexane:ethyl acetate (0.5:3.5) as a solvent. After the product being formed, the reaction mixture was diluted with 70 mL of water and then extracted using 3 × 70 ml of ethyl acetate. The organic phase was collected, washed with 3× 70 ml of water and then dried over anhydrous magnesium sulfate. This organic phase was then evaporated *in vacuo* and the crude product was re-crystallized from a hot methanol to afford the title compound as colourless blocks.

### 2.1. Refinement

N bound H atoms were located from difference Fourier maps and freely refined. The remaining H atoms were positioned geometrically and refined using a riding model with with C–H = 0.95–0.99 Å and U_iso_(H) = 1.2U_eq_(C).

### 3. Results and discussion

The purin ring is almost planar with the maximum deviation of 0.014 (2)Å at atom C11. It makes a dihedral angle of 71.28 and 74.67 (8)° with the two benzene rings, C1—C6 and C14—C19, respectively and these two benzene rings make a dihedral angle of 76.04)10)° with each other (Fig. 1).

In the crytsal structure, two dimers involving N5—H1N5···N3^i^ and C7—H7B···N3^ii^ are observed. These two dimers formed stacked molecules down the *b*-axis. Inter­molecular inter­actions of C8—H8A···N2^iii^ further expand the molecules into infinite layers parallel to the *bc*-plane (Fig. 2).

### Crystal data

**Table:**

C_19_H_17_N_5_S	*F*(000) = 728
*M**_r_* = 347.44	*D*_x_ = 1.420 Mg m^−^^3^
Monoclinic, *P*2_1_/*c*	Mo *K*α radiation, λ = 0.71073 Å
Hall symbol: -P 2ybc	Cell parameters from 3425 reflections
*a* = 16.7346 (7) Å	θ = 2.3–30.0°
*b* = 5.5511 (3) Å	µ = 0.21 mm^−^^1^
*c* = 20.4817 (10) Å	*T* = 100 K
β = 121.325 (3)°	Block, colourless
*V* = 1625.31 (14) Å^3^	0.69 × 0.19 × 0.14 mm
*Z* = 4	

### Data collection

**Table:**

Bruker SMART APEXII CCD diffractometer	4956 independent reflections
Radiation source: fine-focus sealed tube	3416 reflections with *I* > 2σ(*I*)
Graphite monochromator	*R*_int_ = 0.048
φ and ω scans	θ_max_ = 30.6°, θ_min_ = 2.0°
Absorption correction: multi-scan (*SADABS*; Bruker, 2009)	*h* = −23→23
*T*_min_ = 0.868, *T*_max_ = 0.972	*k* = −7→7
16728 measured reflections	*l* = −25→29

### Refinement

**Table:**

Refinement on *F*^2^	Primary atom site location: structure-invariant direct methods
Least-squares matrix: full	Secondary atom site location: difference Fourier map
*R*[*F*^2^ > 2σ(*F*^2^)] = 0.050	Hydrogen site location: inferred from neighbouring sites
*wR*(*F*^2^) = 0.137	H atoms treated by a mixture of independent and constrained refinement
*S* = 1.06	*w* = 1/[σ^2^(*F*_o_^2^) + (0.0602*P*)^2^ + 0.4349*P*] where *P* = (*F*_o_^2^ + 2*F*_c_^2^)/3
4956 reflections	(Δ/σ)_max_ < 0.001
234 parameters	Δρ_max_ = 0.33 e Å^−^^3^
0 restraints	Δρ_min_ = −0.58 e Å^−^^3^

### Special details

**Table:**

Geometry. All e.s.d.'s (except the e.s.d. in the dihedral angle between two l.s. planes) are estimated using the full covariance matrix. The cell e.s.d.'s are taken into account individually in the estimation of e.s.d.'s in distances, angles and torsion angles; correlations between e.s.d.'s in cell parameters are only used when they are defined by crystal symmetry. An approximate (isotropic) treatment of cell e.s.d.'s is used for estimating e.s.d.'s involving l.s. planes.
Refinement. Refinement of *F*^2^ against ALL reflections. The weighted *R*-factor *wR* and goodness of fit *S* are based on *F*^2^, conventional *R*-factors *R* are based on *F*, with *F* set to zero for negative *F*^2^. The threshold expression of *F*^2^ > σ(*F*^2^) is used only for calculating *R*-factors(gt) *etc*. and is not relevant to the choice of reflections for refinement. *R*-factors based on *F*^2^ are statistically about twice as large as those based on *F*, and *R*- factors based on ALL data will be even larger.

### Fractional atomic coordinates and isotropic or equivalent isotropic displacement parameters (Å^2^)

**Table:**

	*x*	*y*	*z*	*U*_iso_*/*U*_eq_	
S1	0.24764 (3)	0.72454 (8)	0.12637 (3)	0.01824 (13)	
N1	0.52964 (10)	1.0733 (3)	0.14394 (8)	0.0147 (3)	
N2	0.42255 (10)	1.0996 (3)	0.17932 (8)	0.0169 (3)	
N3	0.47003 (10)	0.7208 (3)	0.06126 (8)	0.0148 (3)	
N4	0.32973 (10)	0.5624 (3)	0.05291 (8)	0.0151 (3)	
N5	0.39014 (11)	0.3896 (3)	−0.01293 (9)	0.0183 (3)	
C1	0.70179 (12)	0.7950 (3)	0.20967 (10)	0.0170 (4)	
H1A	0.6619	0.7724	0.2293	0.020*	
C2	0.77591 (13)	0.6376 (3)	0.23027 (10)	0.0200 (4)	
H2A	0.7865	0.5080	0.2642	0.024*	
C3	0.83454 (13)	0.6682 (4)	0.20170 (10)	0.0211 (4)	
H3A	0.8838	0.5570	0.2147	0.025*	
C4	0.82082 (13)	0.8621 (3)	0.15408 (10)	0.0210 (4)	
H4A	0.8617	0.8863	0.1355	0.025*	
C5	0.74731 (12)	1.0206 (3)	0.13374 (10)	0.0190 (4)	
H5A	0.7385	1.1538	0.1016	0.023*	
C6	0.68604 (12)	0.9858 (3)	0.16023 (9)	0.0154 (3)	
C7	0.60378 (12)	1.1554 (3)	0.13203 (10)	0.0176 (4)	
H7A	0.6271	1.3124	0.1581	0.021*	
H7B	0.5767	1.1826	0.0767	0.021*	
C8	0.49754 (13)	1.1938 (3)	0.18504 (10)	0.0165 (4)	
H8A	0.5274	1.3330	0.2149	0.020*	
C9	0.46824 (11)	0.8869 (3)	0.10795 (9)	0.0133 (3)	
C10	0.39805 (12)	0.5647 (3)	0.03571 (9)	0.0144 (3)	
C11	0.33280 (12)	0.7292 (3)	0.10083 (9)	0.0138 (3)	
C12	0.40253 (12)	0.9046 (3)	0.13023 (9)	0.0141 (3)	
C13	0.19169 (13)	0.4383 (3)	0.08654 (10)	0.0186 (4)	
H13A	0.2409	0.3215	0.0950	0.022*	
H13B	0.1636	0.3806	0.1160	0.022*	
C14	0.11669 (12)	0.4322 (3)	0.00268 (10)	0.0166 (4)	
C15	0.10259 (13)	0.6128 (3)	−0.04927 (10)	0.0198 (4)	
H15A	0.1421	0.7504	−0.0327	0.024*	
C16	0.03108 (13)	0.5934 (3)	−0.12532 (11)	0.0221 (4)	
H16A	0.0219	0.7190	−0.1601	0.027*	
C17	−0.02712 (13)	0.3932 (3)	−0.15127 (11)	0.0217 (4)	
H17A	−0.0761	0.3812	−0.2033	0.026*	
C18	−0.01235 (13)	0.2113 (3)	−0.09980 (11)	0.0217 (4)	
H18A	−0.0512	0.0726	−0.1168	0.026*	
C19	0.05850 (13)	0.2296 (3)	−0.02383 (11)	0.0196 (4)	
H19A	0.0677	0.1032	0.0107	0.023*	
H1N5	0.4310 (15)	0.370 (4)	−0.0289 (12)	0.030 (6)*	
H2N5	0.3390 (15)	0.304 (4)	−0.0358 (12)	0.024 (6)*	

### Atomic displacement parameters (Å^2^)

**Table:**

	*U*^11^	*U*^22^	*U*^33^	*U*^12^	*U*^13^	*U*^23^
S1	0.0183 (2)	0.0203 (2)	0.0209 (2)	−0.00120 (18)	0.0136 (2)	−0.00239 (18)
N1	0.0136 (7)	0.0158 (7)	0.0139 (7)	−0.0025 (6)	0.0066 (6)	−0.0020 (6)
N2	0.0205 (8)	0.0153 (7)	0.0157 (7)	0.0009 (6)	0.0100 (6)	−0.0001 (6)
N3	0.0160 (7)	0.0152 (7)	0.0150 (7)	−0.0017 (6)	0.0095 (6)	−0.0013 (6)
N4	0.0148 (7)	0.0163 (7)	0.0158 (7)	0.0013 (6)	0.0090 (6)	−0.0001 (6)
N5	0.0161 (8)	0.0203 (8)	0.0209 (8)	−0.0053 (7)	0.0113 (7)	−0.0083 (6)
C1	0.0161 (9)	0.0189 (9)	0.0148 (8)	−0.0039 (7)	0.0072 (7)	−0.0020 (7)
C2	0.0188 (9)	0.0186 (9)	0.0177 (9)	−0.0028 (7)	0.0061 (7)	−0.0005 (7)
C3	0.0174 (9)	0.0218 (9)	0.0200 (9)	0.0004 (7)	0.0067 (8)	−0.0035 (7)
C4	0.0183 (9)	0.0238 (10)	0.0231 (9)	−0.0039 (8)	0.0121 (8)	−0.0046 (8)
C5	0.0203 (9)	0.0188 (9)	0.0176 (8)	−0.0052 (7)	0.0097 (8)	−0.0025 (7)
C6	0.0151 (9)	0.0153 (8)	0.0140 (8)	−0.0039 (7)	0.0062 (7)	−0.0047 (6)
C7	0.0180 (9)	0.0156 (8)	0.0214 (9)	−0.0016 (7)	0.0117 (8)	0.0005 (7)
C8	0.0214 (9)	0.0140 (8)	0.0140 (8)	−0.0004 (7)	0.0092 (7)	−0.0013 (6)
C9	0.0140 (8)	0.0134 (8)	0.0107 (7)	0.0003 (6)	0.0051 (6)	0.0004 (6)
C10	0.0148 (8)	0.0141 (8)	0.0142 (8)	0.0004 (7)	0.0074 (7)	0.0006 (6)
C11	0.0134 (8)	0.0149 (8)	0.0129 (8)	0.0017 (6)	0.0066 (7)	0.0026 (6)
C12	0.0165 (8)	0.0131 (8)	0.0139 (8)	0.0007 (7)	0.0088 (7)	0.0002 (6)
C13	0.0196 (9)	0.0171 (9)	0.0222 (9)	−0.0022 (7)	0.0129 (8)	0.0012 (7)
C14	0.0133 (8)	0.0187 (9)	0.0196 (8)	−0.0001 (7)	0.0099 (7)	−0.0001 (7)
C15	0.0189 (9)	0.0178 (9)	0.0241 (9)	0.0003 (7)	0.0122 (8)	0.0008 (7)
C16	0.0227 (10)	0.0212 (9)	0.0225 (9)	0.0048 (8)	0.0118 (8)	0.0056 (8)
C17	0.0168 (9)	0.0252 (10)	0.0194 (9)	0.0033 (8)	0.0068 (7)	0.0007 (8)
C18	0.0180 (9)	0.0203 (9)	0.0268 (10)	−0.0015 (7)	0.0117 (8)	−0.0013 (8)
C19	0.0184 (9)	0.0187 (9)	0.0235 (9)	0.0003 (7)	0.0122 (8)	0.0017 (7)

### Geometric parameters (Å, º)

**Table:**

S1—C11	1.7551 (17)	C5—C6	1.400 (2)
S1—C13	1.8091 (18)	C5—H5A	0.9500
N1—C9	1.372 (2)	C6—C7	1.513 (2)
N1—C8	1.384 (2)	C7—H7A	0.9900
N1—C7	1.456 (2)	C7—H7B	0.9900
N2—C8	1.307 (2)	C8—H8A	0.9500
N2—C12	1.395 (2)	C9—C12	1.396 (2)
N3—C9	1.340 (2)	C11—C12	1.393 (2)
N3—C10	1.349 (2)	C13—C14	1.512 (2)
N4—C11	1.331 (2)	C13—H13A	0.9900
N4—C10	1.360 (2)	C13—H13B	0.9900
N5—C10	1.348 (2)	C14—C15	1.390 (2)
N5—H1N5	0.90 (2)	C14—C19	1.399 (2)
N5—H2N5	0.87 (2)	C15—C16	1.390 (3)
C1—C2	1.392 (2)	C15—H15A	0.9500
C1—C6	1.392 (2)	C16—C17	1.388 (3)
C1—H1A	0.9500	C16—H16A	0.9500
C2—C3	1.390 (3)	C17—C18	1.386 (3)
C2—H2A	0.9500	C17—H17A	0.9500
C3—C4	1.389 (3)	C18—C19	1.385 (3)
C3—H3A	0.9500	C18—H18A	0.9500
C4—C5	1.388 (3)	C19—H19A	0.9500
C4—H4A	0.9500		
			
C11—S1—C13	100.87 (8)	N3—C9—N1	127.88 (15)
C9—N1—C8	105.78 (14)	N3—C9—C12	126.53 (15)
C9—N1—C7	127.68 (14)	N1—C9—C12	105.59 (14)
C8—N1—C7	125.79 (14)	N5—C10—N3	118.33 (15)
C8—N2—C12	103.57 (14)	N5—C10—N4	114.32 (15)
C9—N3—C10	111.85 (14)	N3—C10—N4	127.34 (15)
C11—N4—C10	117.98 (14)	N4—C11—C12	120.50 (15)
C10—N5—H1N5	123.6 (14)	N4—C11—S1	118.92 (13)
C10—N5—H2N5	118.7 (14)	C12—C11—S1	120.58 (13)
H1N5—N5—H2N5	117.1 (19)	C11—C12—N2	133.42 (15)
C2—C1—C6	120.00 (16)	C11—C12—C9	115.76 (15)
C2—C1—H1A	120.0	N2—C12—C9	110.81 (15)
C6—C1—H1A	120.0	C14—C13—S1	117.49 (13)
C3—C2—C1	120.58 (17)	C14—C13—H13A	107.9
C3—C2—H2A	119.7	S1—C13—H13A	107.9
C1—C2—H2A	119.7	C14—C13—H13B	107.9
C4—C3—C2	119.68 (18)	S1—C13—H13B	107.9
C4—C3—H3A	120.2	H13A—C13—H13B	107.2
C2—C3—H3A	120.2	C15—C14—C19	118.40 (17)
C5—C4—C3	119.92 (17)	C15—C14—C13	124.32 (16)
C5—C4—H4A	120.0	C19—C14—C13	117.28 (16)
C3—C4—H4A	120.0	C16—C15—C14	120.45 (17)
C4—C5—C6	120.63 (17)	C16—C15—H15A	119.8
C4—C5—H5A	119.7	C14—C15—H15A	119.8
C6—C5—H5A	119.7	C17—C16—C15	120.97 (17)
C1—C6—C5	119.13 (16)	C17—C16—H16A	119.5
C1—C6—C7	122.79 (15)	C15—C16—H16A	119.5
C5—C6—C7	118.07 (15)	C18—C17—C16	118.67 (17)
N1—C7—C6	115.28 (14)	C18—C17—H17A	120.7
N1—C7—H7A	108.5	C16—C17—H17A	120.7
C6—C7—H7A	108.5	C19—C18—C17	120.73 (18)
N1—C7—H7B	108.5	C19—C18—H18A	119.6
C6—C7—H7B	108.5	C17—C18—H18A	119.6
H7A—C7—H7B	107.5	C18—C19—C14	120.75 (17)
N2—C8—N1	114.24 (15)	C18—C19—H19A	119.6
N2—C8—H8A	122.9	C14—C19—H19A	119.6
N1—C8—H8A	122.9		
			
C6—C1—C2—C3	−0.2 (3)	C10—N4—C11—C12	2.0 (2)
C1—C2—C3—C4	2.1 (3)	C10—N4—C11—S1	−178.00 (12)
C2—C3—C4—C5	−1.6 (3)	C13—S1—C11—N4	9.65 (15)
C3—C4—C5—C6	−0.6 (3)	C13—S1—C11—C12	−170.34 (14)
C2—C1—C6—C5	−2.0 (2)	N4—C11—C12—N2	178.87 (17)
C2—C1—C6—C7	176.53 (16)	S1—C11—C12—N2	−1.1 (3)
C4—C5—C6—C1	2.4 (2)	N4—C11—C12—C9	−1.9 (2)
C4—C5—C6—C7	−176.18 (16)	S1—C11—C12—C9	178.12 (12)
C9—N1—C7—C6	−69.9 (2)	C8—N2—C12—C11	179.67 (19)
C8—N1—C7—C6	121.46 (18)	C8—N2—C12—C9	0.37 (18)
C1—C6—C7—N1	−14.7 (2)	N3—C9—C12—C11	0.5 (3)
C5—C6—C7—N1	163.78 (15)	N1—C9—C12—C11	−179.30 (14)
C12—N2—C8—N1	−0.76 (19)	N3—C9—C12—N2	179.94 (15)
C9—N1—C8—N2	0.87 (19)	N1—C9—C12—N2	0.13 (18)
C7—N1—C8—N2	171.58 (15)	C11—S1—C13—C14	−83.26 (14)
C10—N3—C9—N1	−179.61 (16)	S1—C13—C14—C15	15.4 (2)
C10—N3—C9—C12	0.6 (2)	S1—C13—C14—C19	−165.09 (13)
C8—N1—C9—N3	179.63 (17)	C19—C14—C15—C16	1.2 (3)
C7—N1—C9—N3	9.2 (3)	C13—C14—C15—C16	−179.25 (16)
C8—N1—C9—C12	−0.55 (17)	C14—C15—C16—C17	−0.6 (3)
C7—N1—C9—C12	−171.03 (16)	C15—C16—C17—C18	−0.3 (3)
C9—N3—C10—N5	178.94 (15)	C16—C17—C18—C19	0.6 (3)
C9—N3—C10—N4	−0.5 (2)	C17—C18—C19—C14	0.0 (3)
C11—N4—C10—N5	179.75 (15)	C15—C14—C19—C18	−0.9 (3)
C11—N4—C10—N3	−0.8 (3)	C13—C14—C19—C18	179.50 (16)

### Hydrogen-bond geometry (Å, º)

**Table:**

*D*—H···*A*	*D*—H	H···*A*	*D*···*A*	*D*—H···*A*
N5—H1*N*5···N3^i^	0.91 (3)	2.14 (3)	3.040 (3)	173 (2)
C7—H7*B*···N3^ii^	0.99	2.57	3.548 (2)	172
C8—H8*A*···N2^iii^	0.95	2.39	3.274 (2)	155

Symmetry codes: (i) −*x*+1, −*y*+1, −*z*; (ii) −*x*+1, −*y*+2, −*z*; (iii) −*x*+1, *y*+1/2, −*z*+1/2.

## References

[bb1] Banh, T. N., Kode, N. R. & Phadtare, S. (2011). *Lett. Drug. Des. Discov.* **8**, 709–716.

[bb2] Bruker (2009). *APEX2*, *SAINT* and *SADABS* Bruker AXS Inc., Madison, Wisconsin, USA.

[bb3] Hadda, V., Pandey, B. D., Gupta, R. & Goel, A. (2009). *JPGM*, **55**, 139–140. 10.4103/0022-3859.52849 19550063

[bb4] Nguyen, T., Vacek, P. M., O’Neill, P., Colletti, R. B. & Finette, B. A. (2009). *Cancer Res.* **69**, 7004–7012. 10.1158/0008-5472.CAN-09-0451 PMC2749269 19706768

[bb5] Salvatore, R. N., Nagle, A. S. & Jung, K. W. (2002). *J. Org. Chem.* **67**, 674–683. 10.1021/jo010643c 11856006

[bb6] Salvatore, R. N., Smith, R. A., Nischwitz, A. K. & Gavin, T. (2005). *Tetrahedron Lett.* **46**, 8931–8935.

[bb7] Sheldrick, G. M. (2008). *Acta Cryst.* A**64**, 112–122. 10.1107/S0108767307043930 18156677

[bb8] Spek, A. L. (2009). *Acta Cryst.* D**65**, 148–155. 10.1107/S090744490804362X PMC2631630 19171970

